# Explaining subjective social status in two countries: The relative importance of education, occupation, income and childhood circumstances

**DOI:** 10.1016/j.ssmph.2021.100864

**Published:** 2021-07-03

**Authors:** Marie Hella Lindberg, Gang Chen, Jan Abel Olsen, Birgit Abelsen

**Affiliations:** a Department of Community Medicine, Faculty of Health Sciences, UiT – the Arctic University of Norway, 9037, Tromsø, Norway; b Centre for Health Economics, Monash University, 15 Innovation Walk, Clayton, VIC, 3800, Australia; c Division for Health Services, Norwegian Institute of Public Health, Marcus Thranes gt. 6, 0473, Oslo, Norway

**Keywords:** Subjective social status, Socioeconomic position, Childhood circumstances, Health inequalities, Australia, Norway

## Abstract

In the literature on social inequalities in health, subjective socioeconomic position (SEP) is increasingly applied as a determinant of health, motivated by the hypothesis that having a high subjective SEP is health-enhancing. However, the relative importance of determinants of subjective SEP is not well understood. Objective SEP indicators, such as education, occupation and income, are assumed to determine individuals' position in the status hierarchy. Furthermore, an extensive literature has shown that past childhood SEP affects adult health. Does it also affect subjective SEP? In this paper, we estimate the relative importance of i) the common objective SEP indicators (education, occupation and income) in explaining subjective SEP, and ii) childhood SEP (childhood financial circumstances and parents' education) in determining subjective SEP, after controlling for objective SEP. Given that the relative importance of these factors is expected to differ across institutional settings, we compare data from two countries: Australia and Norway. We use data from an online survey based on adult samples, with N ≈ 1400 from each country. Ordinary least squares regression is conducted to assess how objective and childhood SEP indicators predict subjective SEP. We use Shapley value decomposition to estimate the relative importance of these factors in explaining subjective SEP. Income was the strongest predictor of subjective SEP in Australia; in Norway, it was occupation. Of the childhood SEP variables, childhood financial circumstances were significantly associated with subjective SEP, even after controlling for objective SEP. This association was the strongest in the Norwegian sample. Only the mother's education had a significant impact on subjective SEP. Our findings highlight the need to understand the specific mechanisms between objective and subjective SEP as determinants of inequalities in health, and to assess the role of institutional factors in influencing these complex relationships.

## Highlights

• Estimates the relative importance of determinants of subjective social status.
• The relative importance of determinants differs between countries.
• Income was the most important in Australia; in Norway it was occupation.
• Childhood circumstances are independently associated with subjective social status.
• Policy should consider how determinants affect subjective social status and health.

## 1 Introduction

In the literature on social inequalities in health, different indicators for socioeconomic position (SEP), most commonly education, occupation and income, are applied (Galobardes et al., 2007). These objective SEP indicators are used to place individuals in the status hierarchy when analysing social inequalities in health. Additionally, an increasingly applied indicator is subjective SEP, also referred to as subjective social status, that reflects how the objective SEP influences individuals’ perceived placement in the social hierarchy (Demakakos et al., 2018; Jackman & Jackman, 1973; Nobles et al., 2013). However, little is known about the relative importance individuals place on these objective SEP indicators when judging their position in society.

A high subjective SEP is hypothesised to be health-enhancing (Marmot, 2004); a range of studies has documented that subjective SEP predicts various health outcomes above and beyond objective SEP measures. The most studied health outcome in this regard is self-rated health (see e.g., Demakakos et al., 2008; Präg, 2020), but subjective SEP has also been found to predict outcomes such as mortality (Demakakos et al., 2018), depression (Singh-Manoux et al., 2003), cortisol levels (Wright & Steptoe, 2005), obesity (Goodman et al., 2003), and vulnerability to the common cold (Cohen et al., 2008).

The hypothesised association between subjective SEP and health inequalities is rooted in psychosocial explanatory pathways (Schnittker & McLeod, 2005). People internalise perceptions of their position in socioeconomic hierarchies through social comparison, which may influence health via neuroendocrine mechanisms related to stress (McEwen & Gianaros, 2010; Nobles et al., 2013). From this perspective, the feeling of inferiority is considered to be a risk factor in itself (Marmot, 2004; Theodossiou & Zangelidis, 2009; Wilkinson, 1999). Subjective SEP is, therefore, both strongly determined by objective SEP, but is also considered a distinct construct, as subjective SEP picks up other aspects than objective SEP in capturing how socioeconomic factors influence health (Demakakos et al., 2008).

Further, extensive literature has established that childhood SEP affects adult health (see e.g., Case et al., 2005; Cohen et al., 2010; Nettle & Bateson, 2017; Smith et al., 1997); however, the many pathways through which this occurs are challenging to trace. The association between childhood and subjective SEP could potentially explain the pathway from childhood SEP to adult health via the status generated from growing up with a ‘silver spoon’. However, the influence of childhood SEP on subjective SEP in adulthood has been sparsely studied (Ferreira et al., 2018; Kim & Radoias, 2019), especially in Western contexts.

The association between childhood and subjective SEP would indicate that not only objective SEP, but also childhood SEP determines subjective SEP: the better the conditions during childhood, the higher the subjective SEP. Nevertheless, the *relative importance* of objective SEP on the one hand and childhood SEP on the other, in determining subjective SEP, remains unknown. Identifying their relative importance is important for developing appropriate policy responses that mitigate the impact of exposure to damaging socioeconomic factors.

The relative importance of determinants of subjective SEP is likely to vary across countries with different macro-level contexts (e.g., economic growth, unemployment rate) and institutional settings (such as social policies), since these factors are likely to shape the determinants of individuals’ subjective SEP. This paper compares Australia and Norway, which have similar life expectancies, and they both rank high on the Human Development Index (Australia 6th, and Norway 1st; UNDP, 2020). In terms of income inequality, measured using the Gini index, Australia (0.33) is more unequal than Norway (0.26) (OECD, 2018a). While both countries have a publicly funded national health service, it is more common in Australia to have voluntary private health insurance (Australian Institute of Health and Welfare, 2020). The share of the population with higher education is similar in the two countries, although it is more common in Norway to pursue postgraduate degrees (10.3% in Norway vs 5.4% in Australia; Australian Bureau of Statistics, 2017; Statistics Norway, 2019).

In this paper, we have quantified respondents' implicit weighting of education, occupation and income in explaining their own subjective SEP, as the relative importance of these factors is not well understood in the assessment of subjective SEP (Navarro-Carrillo et al., 2020). We have further provided new insights into the importance of childhood SEP, measured using childhood financial circumstances and parents’ education level, in determining subjective SEP, to investigate whether there are determinants of adult subjective SEP that can be traced back to early-life conditions, independently of objective SEP.

The aim of this study was to estimate the relative importance of a) objective SEP indicators (education, occupation and income), and b) childhood SEP, independent of objective SEP, in determining subjective SEP in adulthood. Since the relative importance of these components is expected to differ across institutional settings, we have compared data from two countries. The paper is structured as follows: Section 2 describes the data, variables and methods and Section 3 presents the results, followed by a discussion in Section 4 and conclusions in Section 5.

## 2 Material and methods

### 2.1 Data

An anonymous survey was developed on an online survey platform, Qualtrics (www.qualtrics.com). Request responses were set up to increase the question response rate such that respondents were reminded to complete the missing question before moving to the next page, to reduce the number of missing values. The respondents were recruited by Cint (www.cint.com), a global panel company, among members of its panel in December 2018–February 2019. For each country, a targeting sample size of 1400 was used and demographic quotas (with regard to the age and sex distribution) were applied. Initially, a total of 1920 respondents in Australia and 2418 in Norway consented and clicked the survey link. Next, respondents were excluded if they a) did not submit the survey, or the quota was full (N = 249 in Australia; N = 665 in Norway); or b) failed quality thresholds, e.g., spent less than 5 min to complete the survey (N = 248 in Australia; N = 353 in Norway). After the exclusion, the Australian and Norwegian sample sizes were left at N = 1423 and N = 1,400, respectively. Upon completion of the survey, panel members received a small amount of reimbursement for their time and effort to complete the survey. As an example, Cint has successfully facilitated a large multi-instrument comparison study on quality of life and subjective wellbeing across six countries (Richardson et al., 2016).

Post-stratification weights were created after data collection to align the respondent data with population statistics of each country according to age group and sex. The study was approved by the Monash University Human Research Ethics Committee (project ID: 17490).

### 2.2 Variables

The outcome variable, subjective SEP, was measured with the MacArthur scale of subjective social status (Adler et al., 2000), developed to examine how subjective status determines health (Singh-Manoux et al., 2003). The MacArthur scale was originally developed for the US (Adler et al., 2000), but has since been applied in various contexts and populations, making it a frequently applied measure of subjective SEP. The respondents were instructed to place themselves on a ladder with rungs 1–10: ‘Think of the ladder as representing where people stand in society. At the top of the ladder are the people who are best off – those who have the most money, education and the best jobs. At the bottom are the people who are worst off – those who have the least money, least education and the worst jobs or no job. The higher up you are on this ladder, the closer you are to people at the very top, and the lower you are, the closer you are to the bottom’. The variable was analysed as a continuous measure ranging from 1 to 10, with higher values denoting higher subjective SEP.

Education was recorded based on the highest completed of four education levels: primary education up to ten years; upper secondary and vocational school; undergraduate (less than four years of higher education); and postgraduate degree (higher education of four years or more). For the analyses, we used the upper secondary level as the reference due to few respondents in the primary education category in the Norwegian sample.

Income was recorded as the combined gross income of adults in the household, with eight income brackets in the Norwegian sample and ten in the Australian. For the analysis, income was recorded into five categories to approximate similar distributions across income groups for the two samples.

Occupation was grouped into five categories: *not in labour force*; *machinery operators*, *drivers and labourers*; *sales, clerical and service workers*; *technicians and trade workers*; *managers and professionals*. For the analyses, we recoded the occupation variable into three: the categories *not in labour force* and *managers and professionals* were retained, while the other three were merged into the category *other professions*. The category *not in labour force* includes students, unemployed people and people on disability benefits. Retired people were asked to tick the category that best described their latest occupation.

Childhood SEP was measured by factoring in childhood financial circumstances (CFC) and parental education. The CFC variable was recorded as a response to the question: ‘What was your family's financial situation during your childhood?’, with five possible responses: *very good*; *good*, *neither good nor bad*; *difficult*; *very difficult*. As only a few respondents selected *very difficult*, they were included into the category *difficult*. Similar indicators have been used to proxy childhood SEP in a range of epidemiological studies (see e.g., Listl et al., 2018; Straughen et al., 2013). Parents' education was recorded based on the mother's and father's highest completed of four education levels, with the same categorisation as for respondents' own education level. We analysed it by collapsing the higher (post-secondary) education levels into a *tertiary education* category and the primary and upper secondary levels into a *lower than tertiary* category. We dichotomised them due to substantial differences in the distribution of respondents between the Australian and Norwegian samples (e.g., a substantially lower share of respondents with postgraduate degrees among Australian parents than Norwegian ones). Childhood SEP was hypothesised to proxy respondents' degree of social privilege in early life.

We included age as a continuous variable. We also checked for non-linear age terms. Sex was included to investigate sex-specific differences in explaining subjective SEP.

### 2.3 Statistical analysis

#### 2.3.1 Descriptive statistics

Descriptive statistics included means, proportions, and standard deviations reported by country and sex. Missing observations for subjective SEP were excluded from the analyses (N = 0 from the Australian sample; N = 6 from the Norwegian sample). In addition, N = 1 observation was deleted from each of the samples due to the reporting of unlikely high age. This left the Australian sample with N = 1422 respondents, and the Norwegian sample with N = 1393 respondents. The mean subjective SEP scores were presented by education level, income level, occupation category, CFC category and parents’ education level. The difference in subjective SEP scores between Australia and Norway was tested with independent sample t-tests, using 5% as the significance level. The distributions of subjective SEP were displayed using histograms.

#### 2.3.2 Determinants of subjective SEP

Ordinary least squares regression analysis was conducted to assess how the three objective SEP indicators (education, occupation and income) and childhood SEP (CFC and parents’ education) predicted subjective SEP. All analyses were adjusted for age groups and sex. Tests of normally distributed residuals were conducted. Except for age, all other predictors were included as dummies.

We set up three regression models. Model A regressed education and income on subjective SEP, while Model B further included occupation. Model C further included childhood SEP (CFC and parents’ education), referred to as the full model. Wald tests were conducted to assess whether the model coefficients in the two samples were significantly different.

Education and income were analysed separately from occupation because these variables are arguably easier to interpret. As opposed to education (measured in years) and income (measured in money), not all occupation categories can be as easily ordered. Especially in the case of Norway, the various occupational categories are not as clearly linked to a hierarchical understanding of social class as, for example, in the UK. Moreover, the status associated with different occupations are likely to depend on age, since the labour market has radically changed over the past generation. Occupation is also presumably more sensitive to contextual differences. In a comparative setting, we deemed education and income more consistent variables.

We analysed the adult current SEP predictors in the first step because the MacArthur question is framed in terms of the three objective SEP indicators (education, occupation and income), which is in line with other literature studying the relationship between objective and subjective SEP (Andersson, 2018). We then added childhood SEP because we wanted to examine its added importance in explaining subjective SEP, *after* controlling for the three common SEP predictors.

We used Shapley value decomposition to determine the predictor that was the relatively most important for subjective SEP. This is a variance decomposition technique that measures the marginal contribution to the model's explained variance, R^2^, by adding any given predictor variable to the model, weighted by the number of permutations represented by a sub-model that does not contain this predictor (Shorrocks, 2013). The Shapley value therefore reports the value of adding any given predictor to the model as a proportion of R^2^ (Huettner & Sunder, 2012); the larger the value, the greater that variable's relative importance in explaining subjective SEP.

We ran analyses of sex and age interactions with the subjective SEP determinants, as well as sex-stratified analyses. We also tested for interactions between each of the SEP variables. Lastly, we checked whether having a higher education level than any of their parents mattered for their reporting of subjective SEP by adding a dummy for ‘educational mobility’ to Model C.

All statistical analyses were performed with Stata© version 15.1 (Stata Corporation, College Station, Texas). All analyses were conducted using sample weights.

## 3 Results

### 3.1 Descriptive statistics

The sample characteristics are reported in Table 1, with means and standard deviations for continuous variables, and categorical variables as proportions. The appendix Table A.1 provides descriptive statistics of the variables reported in their originally recorded categories.

**Table 1:** Sample characteristics.

Variables	Australia						Norway					
	Female		Male		Total		Female		Male		Total	
	Mean/%	N	Mean/%	N	Mean/%	N	Mean/%	N	Mean/%	N	Mean/%	N
**Age (yrs), mean**	45.4 (15.9)	731	47.0 (17.5)	691	46.2 (16.7)	1422	42.1 (15.2)	566	45.1 (17.9)	833	43.9 (16.9)	1399
(SD)												
**Subjective SEP**	5.6 (1.9)	731	5.9 (2.1)	691	5.8 (2.0)	1422	6.0 (2.1)	566	6.4 (2.0)	827	6.2 (2.1)	1393
(SD)												
**Education level**												
Primary education <10 yrs	27.5	201	24.5	169	26.0	370	8.5	48	5.5	46	6.7	94
Upper secondary	35.2	257	32.7	226	34.0	483	32.9	186	30.7	256	31.6	442
Undergraduate	22.6	165	25.0	173	23.8	338	28.1	159	29.4	245	28.9	404
Postgraduate	14.8	108	17.8	123	16.2	231	30.6	173	34.3	286	32.8	459
**Occupational category**												
Not in labour force	39.1	286	20.7	143	30.2	429	26.2	148	15.6	130	19.9	278
Other professions	34.0	248	41.4	286	37.6	534	49.7	281	52.8	440	51.5	721
Managers & professionals	27.0	197	37.9	262	32.3	459	24.2	137	31.6	263	28.6	400
**Household income in five groups**												
Low	24.5	179	20.7	143	22.6	322	26.2	148	15.3	127	19.7	275
Lower middle	26.1	191	23.4	162	24.8	353	34.3	194	29.7	247	31.5	441
Middle	21.3	156	20.3	140	20.8	296	11.7	66	13.2	110	12.6	176
Upper middle	19.0	139	24.6	170	21.7	309	18.7	106	24.6	205	22.2	311
High	9.0	66	11.0	76	10.0	142	9.2	52	17.3	144	14.0	196
**Childhood financial circumstances**												
Difficult	30.1	220	22.4	155	26.4	375	21.4	121	16.8	140	18.7	261
Neither good nor bad	30.9	226	30.7	212	30.8	438	34.8	197	32.4	270	33.4	467
Good	27.4	200	32.1	222	29.7	422	27.4	155	32.5	271	30.5	426
Very good	11.6	85	14.8	102	13.2	187	16.4	93	18.3	152	17.5	245
**Mother's education**												
≤ Upper secondary	82.5	603	75.8	524	79.3	1127	62.9	356	60.5	504	61.5	860
Tertiary education	17.5s	128	24.2	167	20.8	295	37.1	210	39.5	329	38.5	539
**Father's education**												
≤ Upper secondary	78.8	576	69.5	480	74.3	1056	59.4	336	57.3	477	58.1	813
Tertiary education	21.2	155	30.5	211	25.7	366	40.6	230	42.7	356	41.9	586

*Note*: The undergraduate and postgraduate education levels correspond to university education up to four years, and university education of four years or more, respectively. Standard deviations (SD) in parentheses for continuous variables. The household income groups correspond to the following income brackets in Australia (in AUD): Low: <35,000; Lower middle: 35,001–65,000; Middle: 65,001–100,000; Upper middle: 100,001–160,000; High: >160,001; in Norway (per 1000 NOK): Low: <349; Lower middle: 350–699; Middle: 700–849; Upper middle: 850–1199; High: >1200.

Table 2 displays the mean values of subjective SEP scores for each SEP variable in Australia and Norway, together with the *p* value for the *t*-test of the difference between the two samples’ subjective SEP scores. There was a significant difference between the average subjective SEP score in Australia and Norway, as were the scores for sex. The subjective SEP mean scores were significantly different for nearly all the SEP indicator levels, except for education. For all significant differences, the Norwegian mean SEP scores were higher than the Australian ones.

**Table 2:** Comparisons on subjective SEP scores between Australia and Norway, mean (SD).

	Subjective SEP		
	Australia	Norway	T test *p* value
**Total**	5.8 (2.0)	6.2 (2.1)	***
**Sex**			
Women	5.6 (1.9)	6.0 (2.1)	***
Men	5.9 (2.1)	6.4 (2.0)	***
**Education**			
Primary education <10 yrs	4.9 (2.0)	4.8 (2.4)	
Upper secondary	5.6 (2.0)	5.6 (2.1)	
Undergraduate	6.3 (1.7)	6.3 (1.7)	
Postgraduate	6.8 (2.1)	7.0 (1.9)	
**Household income**			
Low	4.8 (2.1)	5.3 (2.6)	***
Lower middle	5.3 (2.0)	5.9 (2.0)	***
Middle	5.9 (1.7)	6.3 (1.7)	**
Upper middle	6.6 (1.8)	6.7 (1.5)	
High	7.0 (1.7)	7.3 (1.6)	*
**Occupation**			
Not in labour force	4.9 (2.1)	4.9 (2.2)	
Other professions	5.7 (1.9)	6.1 (1.9)	***
Managers & professionals	6.6 (1.8)	7.3 (1.7)	***
**Childhood financial circumstances**			
Difficult	5.2 (2.0)	5.6 (2.3)	***
Neither good nor bad	5.6 (1.9)	5.9 (1.9)	***
Good	6.1 (1.8)	6.3 (1.8)	*
Very good	6.7 (2.5)	7.3 (2.1)	***
**Parents' education**			
Mother: Lower than tertiary	5.6 (2.0)	6.0 (2.0)	***
Mother: Tertiary education	6.6 (2.1)	6.6 (2.1)	
Father: Lower than tertiary	5.5 (2.0)	5.9 (2.0)	***
Father: Tertiary education	6.4 (2.1)	6.6 (2.1)	*

*Note:* *** p < 0.01, ** p < 0.05, * p < 0.1. *p* values were calculated based on independent samples *t*-test, with a 5% significance level. Standard deviations (SD) in parentheses.

The distribution of respondents across the subjective SEP ladder in Australia and in Norway is depicted in Fig. 1a, Fig. 1ba and b respectively. The distribution of subjective SEP scores approximates the normal distribution.

**Fig. 1a:**
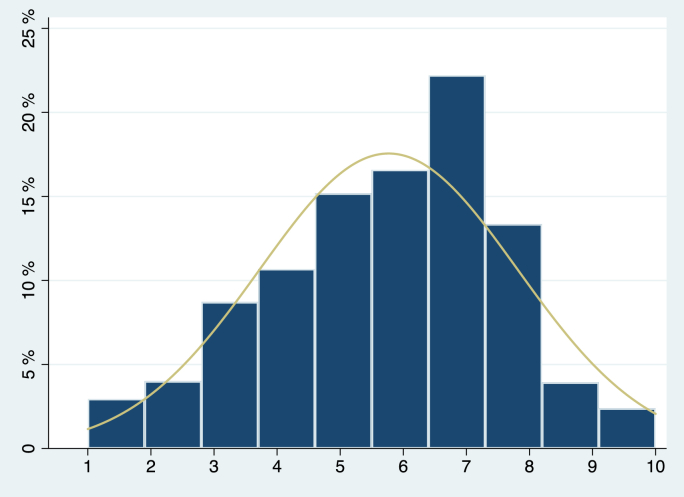
Distribution of respondents across the rungs of the subjective SEP ladder, Australia.

**Fig. 1b:**
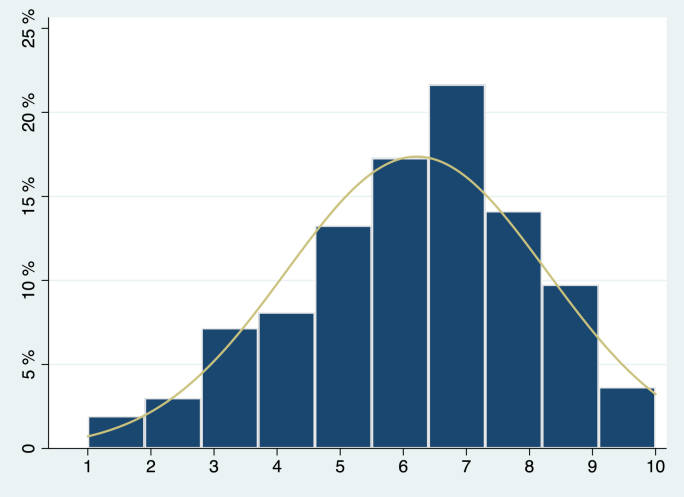
Distribution of respondents across the rungs of the subjective SEP ladder, Norway.

### 3.2 Relative importance of objective indicators in predicting subjective SEP

Table 3 depicts the three regression models. First, education and income, adjusting for age and sex, were regressed on subjective SEP (Table 3, Model A). There was a nearly linear relationship between each increase in income level and subjective SEP, compared with the lowest income category. The association with subjective SEP indicated linearity also for education.

**Table 3:** Ordinary least squares regression results explaining subjective SEP, Australia and Norway.

	A		B		C	
	Australia	Norway	Australia	Norway	Australia	Norway
Education (ref. upper secondary)						
Primary education <10 yrs	−0.51*** (0.14)	−0.86*** (0.24)	−0.45*** (0.14)	−0.66*** (0.24)	−0.45*** (0.13)	−0.63*** (0.24)
Undergraduate	0.43*** (0.13)	0.58*** (0.13)	0.28** (0.13)	0.49*** (0.13)	0.25* (0.13)	0.42*** (0.12)
Postgraduate	0.84*** (0.17)	1.15*** (0.13)	0.58*** (0.17)	0.65*** (0.14)	0.39** (0.17)	0.44*** (0.13)
**Household income** (ref. low)						
Lower middle	0.45*** (0.16)	0.51*** (0.17)	0.41*** (0.16)	0.35** (0.17)	0.47*** (0.15)	0.45*** (0.16)
Middle	0.90*** (0.16)	0.79*** (0.19)	0.77*** (0.16)	0.59*** (0.19)	0.81*** (0.16)	0.69*** (0.18)
Upper middle	1.53*** (0.16)	1.16*** (0.17)	1.35*** (0.17)	0.92*** (0.18)	1.33*** (0.17)	1.04*** (0.17)
High	1.98*** (0.19)	1.64*** (0.18)	1.78*** (0.20)	1.27*** (0.19)	1.75*** (0.19)	1.28*** (0.18)
**Occupation** (ref. other professions)						
Not in labour force			−0.24* (0.13)	−0.74*** (0.16)	−0.21 (0.13)	−0.71*** (0.15)
Managers & professionals			0.42*** (0.13)	0.79*** (0.12)	0.37*** (0.13)	0.62*** (0.12)
**Childhood financial circumstances** (ref. neither good nor bad)						
Difficult					−0.26** (0.12)	−0.06 (0.15)
Good					0.39*** (0.12)	0.38*** (0.11)
Very good					0.74*** (0.20)	1.18*** (0.16)
**Parents' education** (ref. lower than tertiary)						
Mother's tertiary education					0.34** (0.15)	0.30** (0.12)
Father's tertiary education					−0.13 (0.14)	−0.03 (0.12)
**Demographic characteristics**						
Age (yrs.)	0.02*** (0.00)	0.02*** (0.00)	0.01*** (0.00)	0.01*** (0.00)	0.02*** (0.00)	0.02*** (0.00)
Male	0.12 (0.10)	0.03 (0.10)	0.06 (0.10)	−0.02 (0.10)	0.01 (0.10)	−0.08 (0.09)
Constant	4.05*** (0.22)	4.23*** (0.19)	4.30*** (0.24)	4.73*** (0.22)	3.91*** (0.25)	3.97*** (0.24)
*Observations*	*1422*	*1393*	*1422*	*1393*	*1422*	*1393*
*R*^*2*^	*0.19*	*0.21*	*0.20*	*0.26*	*0.23*	*0.30*

*Note:* *** p < 0.01, ** p < 0.05, * p < 0.1. The undergraduate and post-graduate education levels correspond to university education up to four years, and university education of four years or more, respectively. Robust standard errors in parentheses. Sampling weights in both countries included.

In Model B of Table 3, we added occupation, with *other professions* as the reference category. Most of the associations were attenuated compared to Model A. In the Australian sample, the category *not in labour force* was not significant at the 5% level. In the Norwegian sample, there was a strong negative association between being outside of the labour force and subjective SEP, and a strong positive association with subjective SEP for managers and professionals.

Including childhood SEP (Model C) slightly decreased the education coefficients in both samples, whereas income coefficients in the Norwegian sample increased. CFC significantly contributed to the likelihood of reporting a higher subjective SEP in both samples compared to the reference (*neither good nor bad*). In the Norwegian sample, there was no difference in the reporting of subjective SEP for those who reported *difficult* CFC. Respondents who stated *very good* CFC had a subjective SEP of more than one rung higher than the reference. In the Australian sample, the associations of CFC were not as strong, but still made an important contribution in explaining respondents' subjective SEP. CFC contributed more to R^2^ in the Norwegian sample than in the Australian. In the analyses of parents' education, it was only the mother's higher education level that was significant; respondents whose mothers had university education reported 0.34 and 0.30 higher subjective SEP in Australia and Norway, respectively. Father's education was not independently associated with subjective SEP. The reporting of subjective SEP increased with age in all models (Table 3), except for a slight decrease in early adulthood in the Australian sample when adding a quadratic age term (output not reported).

**Fig. 2:**
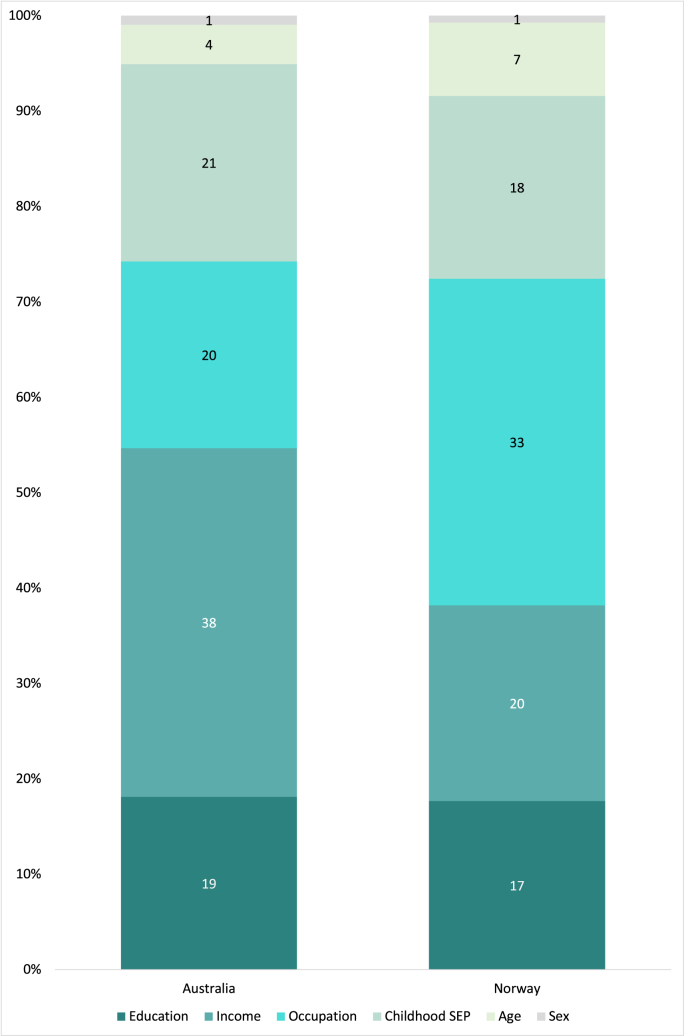
Relative importance of determinants of subjective SEP as a proportion of the R^2^, Australia and Norway, respectively. Each determinant's relative importance is reported as the percentage of the explained variance, R^2^. Based on Shapley value decomposition of Model C in Table 3, in which a 100% corresponds to the models' R^2^ (21% and 26% in the Australian and Norwegian samples, respectively). Determinants of subjective SEP: education, income, occupation and childhood SEP (childhood financial circumstances and parents' education), age and sex.

Shapley value decomposition run on the full model (Table 3, Model C) indicated that income was the most important determinant in the Australian sample, and occupation the most important in the Norwegian. The relative importance of each predictor of subjective SEP is illustrated in Fig. 2, in which each predictor's importance is depicted as a share of the model's R^2^. In this figure, a 100% corresponds to the percentage of total variance explained by the predictors in each country.

Separate analyses conducted with only childhood SEP as predictors of subjective SEP indicated that CFC was independently associated with subjective SEP, as the coefficients were similar to those reported in Model C (output not shown).

Wald tests of the difference between coefficients in the two samples in the full model (Model C) indicated that it was only the coefficients of category *not in labour force* that was significantly different between Australia and Norway (output not shown).

Testing age and sex interactions in Model C identified several significant interaction terms. In the Norwegian sample, there were sex differences across all income levels, where men had an advantage in terms of income-related subjective SEP. The postgraduate education and *very good* CFC coefficients were also significant, favouring women. In the Australian sample, the upper-middle income category interacted with sex. There were significant, positive age interactions for all income levels, and negative age interactions for the two upper education levels and *good* CFC (Appendix Table A.2). Analyses stratified by sex indicated differences in especially the income levels (Appendix Table A.3), in line with the sex interactions from Table A.2. The ‘educational mobility’ dummy added to Model C was not significant (output not reported).

## 4 Discussion

Social inequalities in health are commonly measured using objective SEP indicators, such as individuals' different levels of education, occupation and income. It is claimed that objective indicators ‘produce’ social status, and that people's perceived social status is health-enhancing (Marmot, 2004). However, little is known about the relative importance of these indicators in the subjective assessment of individuals' placement in the social hierarchy (Navarro-Carrillo et al., 2020). Furthermore, the literature has confirmed a lasting impact of childhood SEP on adult health and socioeconomic conditions (Case et al., 2005). It is nevertheless unclear how childhood SEP relates to *subjective* SEP*.* Could it be that childhood SEP also determines adult subjective SEP, through some sort of class consciousness?

In this paper, we have estimated respondents' implicit importance weighting of their own education, occupation and income in explaining subjective SEP, and compared two different countries to assess whether the relative importance of the determinants of subjective SEP vary with the institutional setting. We further expanded the analysis by assessing the relative importance of childhood SEP (CFC and parents' education). We found support for the hypothesis that childhood SEP has a lasting impact on individuals’ subjective SEP, independent of their education, occupation and income, as also reported in Ferreira et al. (2018). Therefore, we theorise that the pathway from childhood SEP to adult health may pass through subjective SEP, independently of adult objective SEP.

The comparison of Australia and Norway suggests some striking differences in the relative importance of subjective SEP determinants between the two countries. In the Australian sample, income was the most important determinant (Fig. 2), possibly attributed to institutional differences, such as income inequality, partly as a result of different redistributive policies. Income inequality is higher in Australia than in Norway, and it is especially the top share of Australian earners that has ‘taken off’ in the past few decades (OECD, 2018b). In Norway, the relatively small income inequalities can be partly explained by a system of collective bargaining between employers and labour unions, ensuring wage coordination and compression across the occupational hierarchy (Barth et al., 2014). The combination of these factors could imply that Australia has larger inequalities in what money do for people's perception of their own SEP.

For example, it is more common in Australia that children from high-income families attend private schools, often associated with prestige and high-quality teaching. Income inequalities could, therefore, directly influence educational inequalities in Australia. This would suggest that the *type* of school seems to be more important than the number of years spent in school. In Norway, private schools are not common, and which school people went to is less likely to influence their subjective SEP. Rather, university-level education is a potential ticket to higher social standing. This is arguably due to Norwegian education policy that incentivises completing upper secondary school by offering universal access to higher education. This has led to an increased uptake of higher education by the population, which over time is likely to dilute the impact of higher education on subjective SEP. Additionally, this has created a highly merit-based education system that could make it increasingly difficult for those who do not have higher education to enter the labour market (Mackenbach, 2012).

In the Norwegian sample, occupation was the most important predictor of subjective SEP. The occupation variable indicated two things: those in managerial positions reported a significantly higher subjective SEP, and being outside of the labour force was a major determinant for lower subjective SEP. This could be linked to the previous point about those without higher education; indeed, respondents with only primary education were overrepresented among those outside of the labour force. A central welfare policy goal in Norway has been to stimulate people to work rather than to provide disability benefits or social security. Policies are, therefore, meant to ensure high labour force participation and advantages to work (Meld. St. 46. (2012–2013)). Our results point to a potentially unintended consequence of this policy: that those who for whatever reason do not work are stigmatised, reflecting negatively on people's perceived SEP. In the Australian sample, those outside of the labour force also reported a lower subjective SEP, but of a smaller magnitude.

Analysing education, occupation and income as predictors of subjective SEP resulted in an R^2^ of 20% and 26% in Australia and Norway respectively (Table 3, Model B). Considering that the MacArthur scale is framed in terms of education, income and occupation, a larger proportion of explained variation could be expected. At the same time, this could limit respondents’ conception of their subjective SEP (Navarro-Carrillo et al., 2020), but our data indicate that respondents included other factors when assessing their subjective SEP. Childhood SEP seems to constitute some of these factors.

In the Australian sample, reports of *difficult* or *very difficult* CFC were significantly associated with lower subjective SEP. In the Norwegian sample, this association was not significant, which could suggest that institutions, such as the school system, provide similar opportunities for children regardless of different social backgrounds. Conversely, those who reported very good CFC had a significantly higher subjective SEP. The magnitude was largest in the Norwegian sample wherein respondents reported more than one rung higher on the subjective SEP ladder. This coefficient was larger than that for the highest income level, suggesting that being raised in prosperous circumstances could contribute to a higher status than living in a high-income household. The *very good* coefficient was smaller among Australian respondents, although significantly higher than the reference. These results could imply that people's subjective SEP is internalised in childhood and that this ‘class consciousness’ remains an integral part of individuals' understanding of where they belong in the social hierarchy. Parents' education had limited independent association with respondents' reporting of subjective SEP, but it still confirmed the importance of mother's education in influencing subjective SEP in adulthood in both samples. This is in line with we found that mother's education was more important than father's, in line with previous findings (see e.g., Chen & Li, 2009).

It should be noted that the CFC question can be perceived and recalled differently according to contextual and cultural factors. However, CFC has previously been found to perform well in proxying childhood SEP when parents’ income records are unavailable (Straughen et al., 2013), and has been widely used (see e.g., Luo & Waite, 2005), also in cross-country studies (Listl et al., 2018). CFC could possibly depend on age, but it was only *good* CFC in the Australian sample that interacted with age. Interaction analyses of sex did however indicate that Norwegian women responding *very good* CFC were more likely to report higher subjective SEP than men.

Other significant interactions for sex in the Norwegian sample was income, favouring men in terms of subjective SEP, potentially due to a larger proportion of men reporting higher income brackets. Highly educated women seemed to benefit more in terms of subjective SEP than men. In the Australian sample, men in the upper-middle income category reported higher subjective SEP than women. Age interacted significantly with the upper education levels and all income categories.

Considering the analysis of age, in the Australian sample, including a quadratic age term to Model C indicated that there was a negative association with subjective SEP for those in early adulthood, but the association turned positive and nearly linear for older respondents. Overall, the results remained largely the same, and for comparison purposes we only kept the linear age term in the main analyses.

The paper's full model explained 23% and 30% of the variance in subjective SEP in Australia and Norway, respectively. As indicated above, there are a range of other potential factors not measured in this survey that could explain subjective SEP. One such factor could be accumulated wealth, which could be an even stronger predictor than occupation and income. This is likely to depend on age, since older people have more accumulated wealth, which could explain why we found that subjective SEP was positively associated with age. This is in line with Andersson (2018), who suggested that wealth was the main predictor of high placements in the ladder, even for those who reported lower average levels of education, occupational prestige and income.

The current paper focuses on adults, but studying *adolescents’* perception of social stratification should be considered in future research, as this is an important development stage in the life course. The youth version of the MacArthur scale is warranted for such analyses (Goodman et al., 2001). In the context of this paper, the relative importance of childhood SEP would probably be greater for adolescents than for adults.

This paper has some limitations. First, there is a risk of selection bias due to the recruitment approach, even if age and sex quotas were applied. Online panels using sample quotas is nevertheless common practice in different health fields (see e.g., Lewis et al., 2016; Johnson et al., 2019), and online panels is considered a cost-effective means to achieve representative samples in a short period of time (Bansback et al., 2014). However, the samples might not be representative in terms of other factors, such as income or education. For example, postgraduates were overrepresented in both samples. Given that this study aimed to investigate the relative importance of each indicator, the overrepresentation of highly educated respondents was not a big concern. Moreover, the distribution of respondents across education levels differed between the two samples: the proportion of respondents with a postgraduate degree was twice as large in Norway as in Australia. This could explain why our data showed a general tendency of lower mean subjective SEP values in Australia than in Norway, since the mean values by the education level were nearly the same (Table 2). Second, we only reported the direct associations of the predictor variables with subjective SEP. Interaction analyses were conducted among the predictors, but we found no systematic tendencies. Interaction analyses of dummy variables are challenging, especially with relatively small samples. Third, the occupation variable is somewhat difficult to interpret, particularly the *not in labour force* category, as it does not distinguish between different reasons for not working. Ideally, this would be split since as different groups as students and unemployed people were analysed in the same group. Fourth, institutional and cultural differences could lead to systematically different interpretations of the subjective SEP question, which we did not account for.

## 5 Conclusions

This study provides new insights into respondents' implicit weighting of objective and childhood factors in predicting subjective SEP. We have estimated the contribution of each of the commonly used objective SEP indicators (education, occupation and income) in explaining subjective SEP. In addition, we have added childhood SEP as an important determinant of subjective SEP; while controlling for the objective SEP variables, we found that the influence of childhood SEP persisted into adulthood. We have further pointed to each of these components’ relative importance in explaining subjective SEP. Lastly, we have demonstrated how the relative contribution of each of these determinants differs between two countries.

As for policy implications, this paper has shed light on the need for intervention in policy areas that would affect subjective SEP, such as reduced income inequalities (Australia) and improved social inclusion policies (Norway). However, considering the ‘subjectiveness’ of the concept, the evidence base for any policy intervention would need to complement findings like these with research on other endpoints, such as well-being and health outcomes.

Future research should further investigate the inconsistency between reported subjective and objective SEP. This could provide information on the characteristics of those who overreport or underreport their subjective SEP, as well as illuminate how subjective SEP is a construct distinct from objective SEP. From a health perspective, more research is needed on the pathway from childhood SEP to health via subjective SEP. Moreover, we need a better understanding of the specific mechanisms between objective and subjective SEP on the one hand, and social inequalities in health on the other, to better grasp the role of subjective SEP as a determinant of health inequalities.

## Financial disclosure statement

The funding sources had no influence on the conduct of this research.

## CRediT authorship contribution statement

**Marie Hella Lindberg:** Conceptualization, Methodology, Software, Validation, Formal analysis, Investigation, Data curation, Roles/. **Gang Chen:** Conceptualization, Methodology, Formal analysis, Writing – review & editing, Resources. **Jan Abel Olsen:** Conceptualization, Writing – review & editing, Funding acquisition, Project administration, Resources, Supervision. **Birgit Abelsen:** Conceptualization, Methodology, Formal analysis, Writing – review & editing, Supervision.

## Declaration of competing interest

None.

## Figures and Tables

**Table A.1:** Descriptive statistics of original data of variables that were collapsed in the regression analyses.

	Australia						Norway						
	**Female**		**Male**		**Total**		**Female**			**Male**		**Total**	
**Variables**	**Mean/%**	**N**	**Mean/%**	**N**	**Mean/%**	**N**	**Mean/%**		**N**	**Mean/%**	**N**	**Mean/%**	**N**
**Mother's education**													
Primary education <10 yrs	60.5	442	56.4	390	58.5	832	35.0		198	37.6	313	36.5	511
Upper secondary	22.0	161	19.4	134	20.8	295	27.9		158	22.9	191	25.0	349
Undergraduate	9.9	72	13.9	96	11.8	168	15.2		86	17.4	145	16.5	231
Postgraduate	7.7	56	10.3	71	8.9	127	21.9		124	22.1	184	22.0	308
**Father's education**													
Primary education <10 yrs	56.1	410	46.9	324	51.6	734	31.3		177	28.9	241	29.9	418
Upper secondary	22.7	166	22.6	156	22.6	322	28.1		159	28.3	236	28.2	395
Undergraduate	13.1	96	17.2	119	15.1	215	14.8		84	18.6	157	17.2	241
Postgraduate	8.1	59	13.3	92	10.6	151	25.8		146	23.9	199	24.7	345
**Occupational category**													
Not in labour force	39.1	286	20.7	143	30.2	429	26.1		148	15.6	130	19.9	278
Machinery operators, drivers & labourers	3.4	25	10.4	72	6.8	97	10.9		62	13.3	111	12.4	173
Sales & service	27.0	197	15.3	106	21.3	303	28.6		162	28.5	237	28.5	399
Technicians & trade workers	3.6	26	15.6	108	9.4	134	10.2		58	11.0	92	10.7	150
Managers & professionals	27.0	197	38.0	263	32.3	460	24.2		137	31.6	263	28.6	400
**Household income AUD**							**Household income per 1000 NOK**						
<25,000	13.8	101	10.0	69	12.0	170	<349	26.1	148	15.3	127	19.6	275
25,001–35,000	10.7	78	10.7	74	10.7	152	350–499	18.0	102	14.5	121	15.9	223
35,001–50,000	15.2	111	12.0	83	13.6	194	500–699	16.2	92	15.1	126	15.6	218
50,001–65,000	10.9	80	11.4	79	11.2	159	700–849	11.6	66	13.2	110	12.6	176
65,001–85,000	11.4	83	10.1	70	10.8	153	850–999	11.6	66	14.7	122	13.4	188
85,001–100,000	10.0	73	10.1	70	10.1	143	1000–1199	7.1	40	10.0	83	8.8	123
100,001–130,000	11.8	86	14.3	99	13.0	185	1200–1399	5.1	29	7.4	62	6.5	91
130,001–160,000	7.3	53	10.3	71	8.7	124	>1400	4.2	24	9.8	82	7.6	106
160,001–220,000	6.6	48	7.8	54	7.2	102							
>220,001	2.5	18	3.3	23	2.9	41							

**Table A.2:** OLS analyses of Model C (Table 3) with age and sex interactions

	Australia		Norway
Education (ref. upper secondary)		Education (ref. upper secondary)	
Primary education <10 yrs	−0.47***">*** (0.13)	Primary education <10 yrs	−0.61**">** (0.23)
Undergraduate	0.96*** (0.33)	Undergraduate	0.42*** (0.13)
Postgraduate	1.32*** (0.46)	Postgraduate	0.75*** (0.17)
**Household income** (ref. low)		**Household income** (ref. low)	
Lower middle	−0.80* (0.48)	Lower middle	0.14 (0.21)
Middle	−0.36 (0.46)	Middle	0.30 (0.27)
Upper middle	−0.31 (0.48)	Upper middle	0.64*** (0.23)
High	−0.35 (0.54)	High	0.97*** (0.24)
**Occupation** (ref. other professions)		**Occupation** (ref. other professions)	
Not in labour force	−0.27** (0.13)	Not in labour force	−0.69*** (0.16)
Managers & professionals	0.34*** (0.13)	Managers & professionals	0.58*** (0.12)
**Childhood financial circumstances** (ref. neither good nor bad)		**Childhood financial circumstances** (ref. neither good nor bad)	
Difficult	−0.28** (0.12)	Difficult	−0.03 (0.15)
Good	1.43*** (0.32)	Good	0.36*** (0.11)
Very good	0.78*** (0.20)	Very good	1.67*** (0.22)
**Parents' education** (ref. lower than tertiary)		**Parents' education** (ref. lower than tertiary)	
Mother's tertiary education	0.33** (0.15)	Mother's tertiary education	0.31** (0.12)
Father's tertiary education	−0.20 (0.14)	Father's tertiary education	−0.01 (0.12)
**Demographic characteristics**		**Demographic characteristics**	
Age (yrs.)	0.01* (0.01)	Age (yrs.)	0.02*** (0.00)
Male	−0.15 (0.12)	Male	−0.51* (0.26)
**Interactions**		**Interactions**	
Age X Undergraduate education	−0.02** (0.01)	Male X Postgraduate education	−0.55*** (0.19)
Age X Postgraduate education	−0.02** (0.01)	Male X Lower middle income	0.83** (0.31)
Age X Lower middle income	0.02*** (0.01)	Male X Middle income	1.01*** (0.36)
Age X Middle income	0.02*** (0.01)	Male X Upper middle income	1.01*** (0.31)
Age X Upper middle income	0.03*** (0.01)	Male X High income	0.96*** (0.34)
Age X High income	0.03*** (0.01)	Male X Very good CFC	−0.91*** (0.27)
Age X Good CFC	−0.02*** (0.01)		
Male X Upper middle income	0.53** (0.22)		
Constant	4.39*** (0.41)	Constant	4.07*** (0.27)
*Observations*	*1422*	*Observations*	*1393*
*R*^*2*^	*0.25*	*R*^*2*^	*0.32*

*Note:* *** p < 0.01, ** p < 0.05, * p < 0.1. There were no significant interactions with age in the Norwegian sample. The undergraduate and post-graduate education levels correspond to university education up to four years, and university education of four years or more, respectively. CFC: childhood financial conditions. Robust standard errors in parentheses. Sampling weights in both countries included.

**Table A.3:** Ordinary least squares analyses of Model C (Table 3) stratified by sex

	Australia		Norway	
	Female	Male	Female	Male
Education (ref. upper secondary)				
Primary education <10 yrs	−0.42** (0.18)	−0.49** (0.21)	−0.53 (0.33)	−0.74** (0.32)
Undergraduate	0.38** (0.17)	0.17 (0.19)	0.61*** (0.20)	0.20 (0.16)
Postgraduate	0.20 (0.23)	0.54** (0.25)	0.75*** (0.21)	0.13 (0.17)
**Household income** (ref. low)				
Lower middle	0.39** (0.20)	0.57** (0.25)	0.06 (0.22)	1.06*** (0.24)
Middle	0.74*** (0.20)	0.84*** (0.25)	0.20 (0.27)	1.43*** (0.25)
Upper middle	1.03*** (0.22)	1.60*** (0.26)	0.52** (0.24)	1.77*** (0.23)
High	1.50*** (0.27)	1.93*** (0.29)	0.84*** (0.25)	2.06*** (0.26)
**Occupation** (ref. other professions)				
Not in labour force	−0.05 (0.16)	−0.41* (0.22)	−0.86*** (0.23)	−0.41** (0.20)
Managers & professionals	0.60*** (0.19)	0.20 (0.17)	0.71*** (0.18)	0.51*** (0.16)
**Childhood financial circumstances** (ref. neither good nor bad)				
Difficult	−0.10 (0.16)	−0.46** (0.19)	0.06 (0.23)	−0.08 (0.18)
Good	0.26 (0.17)	0.51*** (0.17)	0.32* (0.17)	0.39*** (0.15)
Very good	0.89*** (0.26)	0.63** (0.30)	1.67*** (0.24)	0.71*** (0.21)
**Parents' education** (ref. lower than tertiary)				
Mother's tertiary education	0.32 (0.20)	0.35 (0.21)	0.39** (0.18)	0.28 (0.17)
Father's tertiary education	0.04 (0.20)	−0.30 (0.19)	−0.12 (0.18)	0.09 (0.15)
Age (yrs.)	0.02*** (0.00)	0.02*** (0.00)	0.02*** (0.01)	0.02 (0.00)
Constant	3.67*** (0.32)	4.13*** (0.39)	3.98*** (0.38)	3.66*** (0.29)
*Observations*	*731*	*691*	*566*	*827*
*R*^*2*^	*0.22*	*0.25*	*0.38*	*0.27*

*Note:* *** p < 0.01, ** p < 0.05, * p < 0.1. The undergraduate and post-graduate education levels correspond to university education up to four years, and university education of four years or more, respectively. Robust standard errors in parentheses. Sampling weights in both countries included.
